# Selection of Host Plasma Membrane Lipids by HIV‐1 Gag Protein

**DOI:** 10.1002/bies.70090

**Published:** 2025-11-20

**Authors:** Nario Tomishige, Yves Mély, Toshihide Kobayashi

**Affiliations:** ^1^ Laboratoire De Bioimagerie et Pathologies UMR 7021 CNRS Faculté De Pharmacie Université De Strasbourg Illkirch France; ^2^ Cellular Informatics Laboratory RIKEN CPR Wako Saitama Japan; ^3^ Holo‐Bio Co. Ltd. Goryo‐Ohara Kyoto Japan; ^4^ Kyoto University of Advanced Sciences Kyoko Japan

**Keywords:** cholesterol, human immunodeficiency virus type 1, lipid asymmetry, lipid domains, lipid imaging, phosphatidylinositol‐4,5‐bisphosphate, sphingomyelin

## Abstract

Human immunodeficiency virus type 1 (HIV‐1) possesses an envelope enriched with a specific set of host plasma membrane (PM) lipids, a composition that is critical for viral infectivity. Virus budding is initiated by the binding of the viral Gag protein to phosphatidylinositol‐4,5‐bisphosphate (PI(4,5)P_2_) located in the inner leaflet of the PM. However, the mechanism by which inner leaflet‐associated Gag protein contributes to the enrichment of specific outer leaflet lipids, such as sphingomyelin (SM) and cholesterol (Chol), remains poorly understood. Visualization of endogenous lipids using specific lipid probes and advanced microscopy has revealed that Gag multimerization reorganizes SM‐ and Chol‐rich lipid domains in a curvature‐dependent manner. To further elucidate the molecular mechanisms underlying Gag‐induced selective lipid enrichment across the bilayer, two potential scenarios are discussed: one involving interdigitation and the other involving Chol enrichment through flip‐flop. These models are considered in the context of existing literature describing the distribution and interactions of SM, PI(4,5)P_2_, and Chol within the PM.

## Graphical abstract

Enrichment of sphingomyelin in the HIV‐1 envelope arises from Gag binding to PI(4,5)P_2_, which triggers reorganization of plasma membrane sphingomyelin/cholesterol domains through unknown mechanisms. We propose that cholesterol‐mediated interleaflet coupling between sphingomyelin and PI(4,5)P_2_, rather than interdigitation, underlies Gag‐induced lipid reorganization.

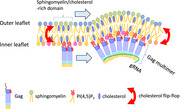

## 1 HIV‐1 Envelope is Enriched in Sphingomyelin, Cholesterol, and Phosphatidylinositol‐4,5‐Bisphosphate

Human immunodeficiency virus type 1 (HIV‐1) is an enveloped virus in which the viral RNA and proteins are surrounded by a membrane composed of proteins and lipids. As the viral genome does not encode enzymes for lipid synthesis, HIV‐1 must “hijack” lipids from the host cell. Infection begins with the fusion of viral particles to the host PM, followed by the release of the cone‐shaped capsid into the cytoplasm, reverse transcription of viral RNA into DNA, and integration of this DNA into the host genome. Subsequent transcription of viral genes leads to the production of viral proteins in the cytoplasm, using viral RNA exported from the nucleus. Viral proteins and genomic RNA then assemble at the PM [1]. The final step of the HIV‐1 lifecycle is budding from the host cell surface, during which the virus acquires its lipid envelope from the PM.

**FIGURE 1:**
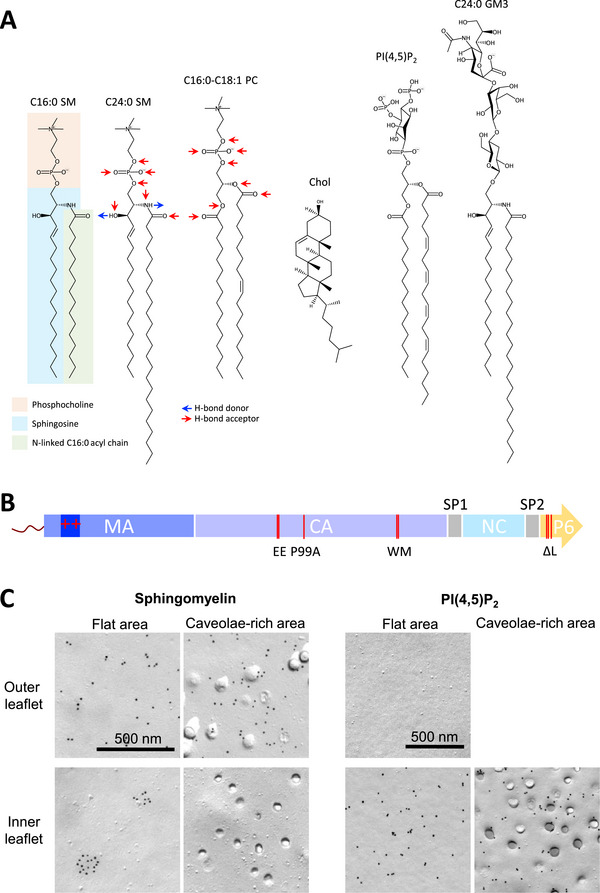
Lipid structure and transbilayer distribution of SM and PI(4,5)P_2_ in the plasma membrane of human skin fibroblasts. **(A)** Chemical structures of representative lipids found in the plasma membrane, shown from left to right: C16:0 SM, C24:0 SM, 16:0‐18:1 PC, Chol, C18:0‐C20:4 PI(4,5)P_2_, and C24:0 GM3. In the C16:0 SM structure, the phosphocholine head group, sphingosine backbone, and C16:0 acyl chain are highlighted by orange, blue, and green boxes, respectively. Hydrogen‐bond‐donating and accepting groups are indicated by blue and red arrows for C24:0 SM and 16:0‐18:0 PC, respectively. **(B)** Domain organization of the HIV‐1 Gag polyprotein. From the N‐terminus: MA, CA, NC, and P6 domains along with spacer peptides SP1 and SP2. N‐terminal myristylation occurs at the glycine residue. The highly basic region (HBR) within MA is indicated by a dark blue box with red “++”. Sites of mutations associated with defective Gag multimerization (WM), impaired curvature formation (EE and P99A), and defective membrane budding (ΔL) are marked with red bars [58]. **(C)** Transbilayer distribution of SM and PI(4,5)P_2_ in the plasma membrane of human skin fibroblasts. SM was labeled using the SM‐specific binding toxin, lysenin (Lys), while PI(4,5)P_2_ was detected using a specific monoclonal antibody [60]. Quantitative imaging revealed that 88% of Lys‐positive signal was localized to the outer leaflet, whereas 99% of PI(4,5)P_2_‐positive signal was restricted to the inner leaflet. Notably, SM in the inner leaflet forms discrete lipid domains in flat regions of the membrane, but not in caveolae‐PM invaginations enriched in SM and cholesterol. Adapted from [60].

The lipid composition of the HIV‐1 envelope is critical for maintaining envelope integrity during viral trafficking and for mediating efficient fusion with the next target cell [2, 3, 4, 5, 6, 7, 8, 9]. Notably, the lipid composition of the viral envelope differs significantly from that of the producer cell's PM. HIV‐1 particles are enriched in sphingomyelin (SM), cholesterol (Chol), ganglioside GM3, and phosphatidylinositol‐4,5‐bisphosphate (PI(4,5)P_2_), and are comparatively depleted in phosphatidylinositol and unsaturated phosphatidylcholine (PC) [3, 10, 11, 12, 13] (Figure 1A). SM, a sphingophospholipid enriched in the outer leaflet of the PM (∼15% of total phospholipids [14, 15]), consists of phosphorylcholine headgroup attached to ceramide, a bioactive lipid. GM3 is a glycosphingolipid (ganglioside) containing a single sialic acid moiety linked to the terminal galactose group of lactosylceramide. PI(4,5)P_2_ is a major phosphoinositide at the PM (1%–2% of PM lipids [16]) and carries phosphomonoesters at positions 4 and 5 of the inositol ring. It serves as a key signaling lipid by recruiting proteins via its anionic head group and acting as a precursor of second messengers, such as inositol triphosphates and diacylglycerol [17, 18, 19]. PI(4,5)P_2_ binds a number of physiologically important intracellular proteins, modulating their localization and function [16, 20]. Chol is typically intercalated within membranes, with its 3‐hydroxyl group positioned near the membrane‐aqueous interface and its flat, rigid four‐ring structure aligned with the acyl chains of neighboring lipids. PC is a glycerophospholipid that shares the same phosphocholine headgroup as SM but has a diacylglycerol backbone. PC constitutes roughly 50% of total PM phospholipids [15, 21] (Figure 1A).

These lipids are not randomly distributed in the PM. Instead, they form specific lipid domains, implying that HIV‐1 either buds from pre‐existing microdomains or actively induces the formation of such domains. Lipid domains enriched in SM and Chol are known as lipid rafts [22, 23, 24]. Lipid rafts are postulated to be involved in a number of physiological and pathological events as diverse as signal transduction to membrane traffic [24, 25]. Because SM/Chol complexes exhibit low solubility in non‐ionic detergents [26, 27, 28], lipid rafts were initially identified as detergent‐insoluble membrane fraction [29]. This biochemical property was exploited in early studies to investigate the involvement of lipid rafts in HIV budding [30, 31, 32, 33, 34, 35, 36, 37, 38]. However, given that detergents can artificially reorganize lipid domains [28, 39, 40, 41], detergent‐free approaches are necessary for studying lipid dynamics during the viral assembly and budding process. Recent advances in specific lipid‐binding probes and high‐resolution quantitative microscopy have enabled in situ visualization of lipid reorganization during HIV‐1 Gag assembly.

Although the specific lipid composition of the HIV‐1 envelope is well characterized, the molecular mechanisms by which these lipids are selectively enriched from the host cell PM remain incompletely understood. A variety of host and viral proteins contribute to the assembly and budding of HIV‐1 [1]. However, the minimal viral component required to drive budding at the PM is the Gag protein, whose expression alone is sufficient to induce the formation of virus‐like particles (VLPs) bearing a lipid envelope derived from the host cell membrane [42, 43, 44].

Gag is synthesized in the cytoplasm as a 55 kDa polyprotein composed of multiple domains: matrix (MA), capsid (CA), spacer peptide 1 (SP1), nucleocapsid (NC), SP2, and P6 (arranged from the N‐ to C‐terminus). The polyprotein is cleaved into individual proteins during viral maturation following budding [1] (Figure 1B). In the cytoplasm, Gag binds RNA, promoting its oligomerization [45, 46, 47, 48]. The resulting oligomers are then targeted to the inner leaflet of the PM, where they bind to PI(4,5)P_2_ [49]. This membrane targeting is mediated by both a myristoyl moiety and a basic amino acid‐rich motif within the MA domain [50].

Upon membrane association, Gag multimerizes to form a two‐dimensional lattice structure, which acts as a scaffold for viral assembly. Chol also plays a critical role in this process: its presence enhances Gag binding to model membranes containing PI(4,5)P_2_ [51, 52, 53], while Chol depletion inhibits HIV production [32] by reducing Gag binding to the PM and impairing Gag multimerization [54]. As Gag multimerization proceeds, the lattice induces membrane curvature, initiating the formation of viral bud. Thus, Gag assembly is spatially restricted to lipid microdomains within the inner leaflet of the PM that are enriched in both PI(4,5)P_2_ and Chol. The final budding event is mediated by host cellular machinery, including the endosomal sorting complexes required for transport (ESCRT) through the interaction with Gag p6 domain [55, 56]. Although Gag assembly has been shown to reorganize SM‐rich lipid domains in the PM [57, 58], the precise lipid composition of HIV‐1 Gag‐only VLPs has not yet been fully characterized.

## 2 Transbilayer Asymmetry and Lateral Heterogeneity of Plasma Membrane Lipids

In the PM of mammalian cells, lipids are asymmetrically distributed between the two leaflets of the bilayer. PI(4,5)P_2_, phosphatidylethanolamine (PE), and phosphatidylserine (PS) are predominantly localized to the inner (cytoplasmic) leaflet while PC, SM, and glycolipids are mainly found in the outer (extracellular) leaflet [59, 60, 61] (Figure 1C). The transbilayer distribution of Chol, however, remains a topic of ongoing debate [62]. Considering the high concentration of Chol (∼40 mol%) in the PM bilayer [21, 62, 63] and the solubility of Chol in the membrane reaches around 60 mol% [64], Chol is unlikely to be restricted in one leaflet.

In addition to the transbilayer asymmetry, lipids within the PM exhibit lateral heterogeneity, forming distinct microdomains. SM, in combination with Chol, can organize into specific lipid domains with estimated diameters ranging from 5 to 50 nm [65, 66, 67, 68, 69]. Structurally, SM consists of a long‐chain base (typically sphingosine) that is amide‐linked to either long‐chain (e.g., C16:0 [indicating the number of carbon (16) and double bond (0) in the fatty acid moiety] or C18:0) or very long‐chain (e.g., C24:0 or C24:1) fatty acid, and contains a phosphocholine head group [70]. Compared to major phospholipids such as PC, which consists of a phosphocholine headgroup and typically one saturated and one unsaturated fatty acid (Figure 1A), SM is more rigid and exhibits a higher gel‐to‐liquid crystalline phase transition temperature [71]. Importantly, SM, especially the C16:0 SM, has a high affinity for Chol [72, 73, 74, 75], whereas PC with unsaturated acyl chains interacts only weakly with Chol. This differential affinity underlies the formation of tightly packed, cholesterol‐rich lipid domains, known as liquid‐ordered (Lo) phases, commonly referred to as lipid rafts. These Lo domains are laterally segregated from more loosely packed, unsaturated PC‐rich liquid‐disordered (Ld) domains [76, 77, 78]. Chol also exerts an ordering effect on Ld membranes, and unsaturated PCs can exhibit Lo‐like behavior when mixed with Chol in binary membrane systems [79, 80].

Given that the diameter of an HIV‐1 particle is approximately 100–150 nm [81, 82, 83, 84], the membrane area required for its formation spans roughly 200–300 nm in diameter. Therefore, it is unlikely that a single small Lo domain (5–50 nm) is sufficient to serve as the sole site for viral assembly and budding. Instead, HIV‐1 assembly likely involves the lateral recruitment and coalescence of multiple small Lo domains into a larger membrane area at the site of Gag assembly [5]. Supporting this model, Gag has been shown to promote the coalescence of lipid raft domains and tetraspanin‐enriched microdomains (TEMs) [85]. TEMs are specialized membrane microdomains enriched in tetraspanins, a family of proteins with four transmembrane domains, and their associated partner proteins. These domains have been implicated in processes such as virus entry, assembly, and budding [86, 87]. However, despite these observations, the mechanisms by which Gag reorganizes and integrates these lipid and protein microdomains remain poorly understood.

## 3 Gag Induces Re‐Distribution of Endogenous SM‐Rich and Chol‐Rich Domains

Recently, two groups investigated the dynamics of SM during Gag assembly using fluorescent SM analogs. Sengupta et al. demonstrated the enrichment of NBD‐C6‐SM at Gag assembly sites using total internal reflection fluorescence (TIRF) microscopy [57]. In contrast, Favard et al. reported that Gag did not confine ATTO 647N‐labeled SM at the assembly site, based on super‐resolution fluorescence correlation spectroscopy (FCS) [88]. This discrepancy may be attributed to the differing physical properties of the fluorescent SM analogs used in each study. A comparative analysis of these analogs in model membrane systems, including giant unilamellar vesicles (GUVs) and giant plasma membrane (PM) vesicles (GPMVs), revealed that NBD‐C6‐SM partitions into liquid‐ordered (Lo) phases at rates of approximately 13% in GUVs and 46% in GPMVs. In contrast, ATTO 647N‐SM exhibits much lower partitioning, with only 4% in GUVs and 18% in GPMVs [89]. These findings underscore the importance of following endogenous lipids when studying domain behavior in live cells. They also suggest that Gag's influence on SM‐rich domain reorganization may be more pronounced in Lo‐phase‐preferring lipids, highlighting a potential mechanism by which Gag selectively remodels membrane microdomains during viral assembly.

**FIGURE 2:**
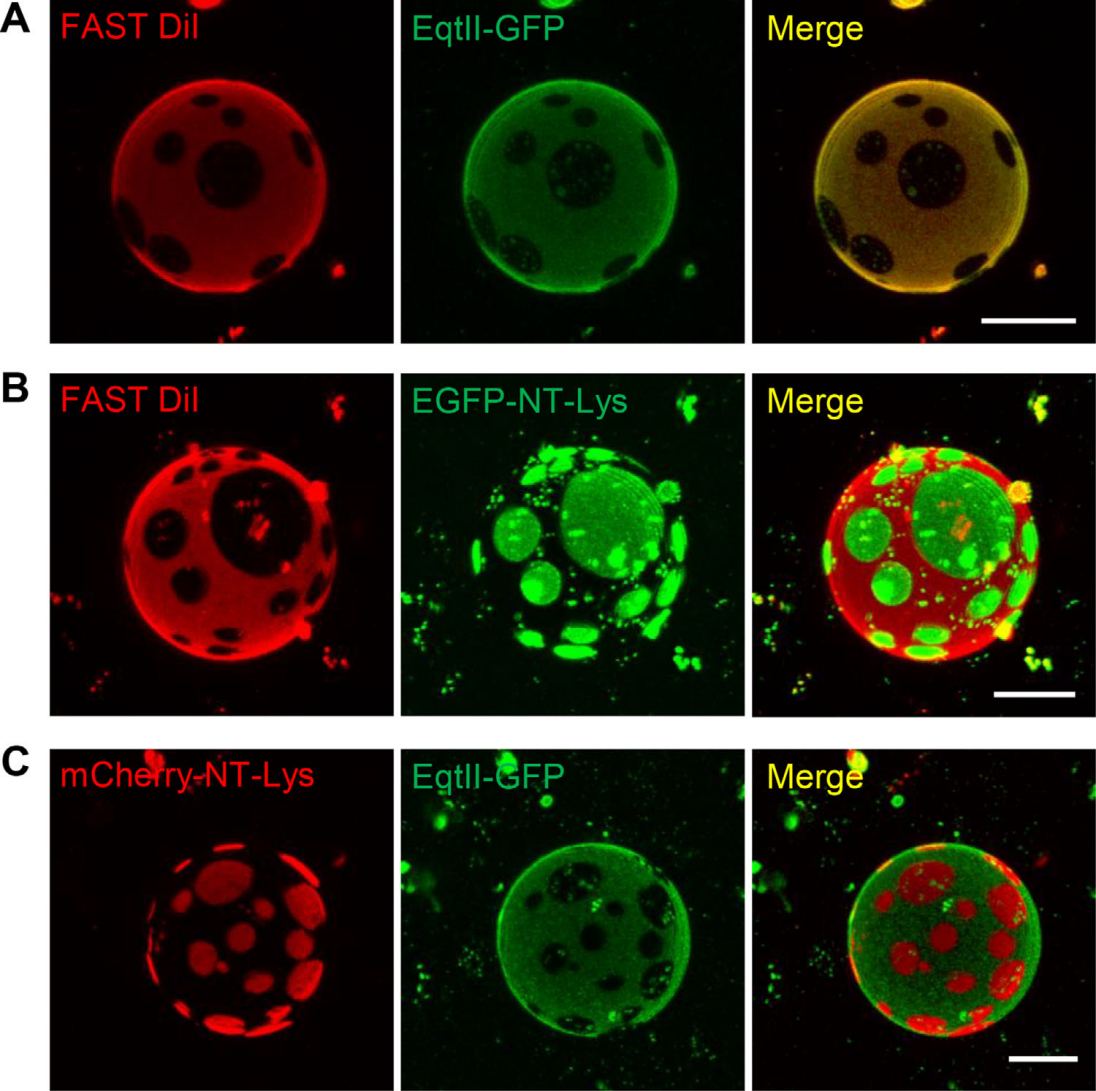
SM distributes in both Lo and Ld domains in model membranes. (**A**) EqtII‐GFP was added to GUVs composed of egg SM/diC18:1 PC/Chol at 2:2:1 molar ratio, along with the Ld phase marker, FAST DiI. EqtII‐GFP colocalized with the Ld phase. (**B**) EGFP‐NT‐Lys was added to GUVs of the same lipid composition. EGFP‐NT‐Lys was excluded from Ld regions (FAST DiI‐positive) and localized to Lo domains. (**C**) GUVs were co‐labeled with mCherry‐NT‐Lys and EqtII‐GFP. Confocal fluorescence microscopy revealed distinct localization of NT‐Lys and EqtII, confirming their preferential binding to Lo and Ld domains, respectively. Scale bars, 20 µm. Adapted from [95].

Recent advances in SM‐specific binding proteins have enabled the direct visualization of endogenous SM in biological membranes [90]. Two such tools—lysenin (Lys), derived from the earthworm *Eisenia fetida*, and equinatoxin II (EqtII), from the sea anemone *Actinia equina*—have been widely employed to study SM distribution and organization [91, 92, 93]. In Figure 2, phase‐separated liposomes composed of egg SM (primarily C16:0 SM)/1,2‐dioleoyl‐*sn*‐glycerophosphocholine (diC18:1 PC)/ and Chol at a molar ratio of 2:2:1 were used to examine the binding specificity of EqtII and a non‐toxic mutant of lysenin (NT‐Lys). EqtII colocalized with the Ld marker, FAST DiI, while NT‐Lys was excluded from Ld regions and selectively bound to Lo domains. This differential localization indicates distinct binding preferences: Lysenin recognizes clusters of 5–6 SM molecules, while EqtII binds to SM monomers [94, 95].

These results, along with prior findings [96, 97], suggest that NT‐Lys binds to SM clusters preferentially located in Lo domains, whereas EqtII targets monomeric SM dispersed in Ld regions. This implies that SM is capable of partitioning into both Lo and Ld phases, but adopts distinct organizational states: clustered in Lo domains and dispersed in Ld domains. These observations not only validate the utility of SM‐binding proteins in domain‐specific lipid studies but also support the model of lipid domain heterogeneity, in which the biophysical state of SM differs depending on the membrane phase context.

We used NT‐Lys to visualize the dynamics of endogenous SM‐rich lipid domains during Gag assembly [58, 98]. Studies using model membranes have shown that NT‐Lys binds to pre‐existing clusters of SM, but does not induce SM clustering on its own [92]. Therefore, a lack of NT‐Lys binding should be interpreted as indicative of the absence of SM clusters, rather than the absence of SM itself [99]. SM clustering is highly dependent on the lipid environment, particularly the presence of glycolipids, which influence SM packing and domain formation [94]. Therefore, careful biochemical analysis of lipid composition, in conjunction with lipid imaging and appropriate control experiments, is essential for accurate interpretation of SM domain dynamics in living cells.

Super‐resolution microscopy, photoactivated localization microscopy (PALM)/direct stochastic optical reconstruction microscopy (dSTORM), has revealed transbilayer co‐localization between inner leaflet Gag and outer leaflet SM‐rich lipid domains in the PM. PALM/dSTORM further showed that the size of SM‐rich domains increases during Gag assembly, suggesting Gag‐induced lipid domain remodeling. Complementary fluorescence recovery after photobleaching (FRAP) experiments demonstrated that Gag expression increases the immobile fraction of SM‐rich lipid domains, consistent with the formation of more stable or clustered lipid platforms. Moreover, fluorescence lifetime imaging microscopy based on Förster resonance energy transfer (FLIM‐FRET) revealed that Gag induces reorganization both among SM‐rich lipid domains and between SM‐rich and Chol‐rich domains [58].

**FIGURE 3:**
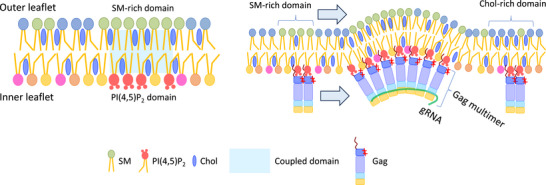
Model of transbilayer lipid domain coupling and Gag‐induced lipid reorganization. **(Left)** Schematic model illustrating a pre‐existing, coupled lipid domain spanning both leaflets of the PM. In this model, SM‐rich domains in the outer leaflet are spatially aligned with PI(4,5)P_2_‐rich domains in the inner leaflet, forming a transbilayer lipid platform. Such coupling may be mediated by lipid‐lipid interactions, interleaflet coupling mechanisms, or membrane curvature. **(Right)** Upon expression and multimerization of HIV‐1 Gag, PI(4,5)P_2_ in the inner leaflet is bound and clustered by the matrix (MA) domain of Gag. This leads to the reorganization and coalescence of SM‐rich lipid domains in the outer leaflet, likely through curvature‐induced stabilization and transbilayer communication. The resulting enlarged and more stable lipid domains may facilitate efficient viral assembly and budding.

Importantly, this lipid reorganization appears to be curvature‐dependent, suggesting that membrane curvature generated during Gag multimerization helps stabilize lipid domain architecture [58, 100]. Supporting this, dSTORM imaging revealed increased colocalization between Alexa Fluor 647 (AF647)‐SNAP‐labeled NT‐Lys (a probe for SM clusters) and Janelia Fluor 549‐SNAP‐D4 (a cholesterol‐binding probe) upon Gag expression [58]. Together, these findings suggest that HIV‐1 Gag binds PI(4,5)P_2_ in the inner leaflet of the PM and induces reorganization of outer leaflet SM, likely due to transbilayer coupling between inner leaflet PI(4,5)P_2_ and outer leaflet SM. This coupling may facilitate the formation of stable, virus‐permissive membrane domains during HIV‐1 assembly (Figure 3).

## 4 Transbilayer Co‐Localization of SM‐Rich Domains and PI(4,5)P_2_ Domains

In model membranes, lipids in the outer and inner leaflets can communicate with each other through hydrophobic interactions involving saturated long fatty acid chains—a phenomenon known as interdigitation [25, 101, 102, 103, 104]. Interdigitation of acyl chains in glycosylphosphatidylinositol (GPI)‐anchored PM proteins and C18 PS has also been suggested [105]. SM, enriched in saturated very long‐chain fatty acids, is particularly well‐suited to forming interdigitated structures. In contrast, PI(4,5)P_2_ typically contains one saturated long‐chain fatty acid (mainly stearic acid, C18:0) and one unsaturated or polyunsaturated fatty acid.

Using synthetic PI(4,5)P_2_ with short acyl chains, Saad et al. proposed a model in which the unsaturated acyl chain of the PI(4,5)P_2_ flips into a hydrophobic pocket within the Gag MA domain during the interaction with Gag. Simultaneously, the saturated myristoyl group of Gag is inserted into the lipid membrane [106]. The removal of the unsaturated chain from the PM alters the physical properties of PI(4,5)P_2_, enabling the Gag/PI(4,5)P_2_ complex to partition into SM‐rich domains. Although this model is compelling, it does not appear to occur with naturally acylated PI(4,5)P_2_ species [107, 108].

Consequently, it remains an open question whether Gag targets pre‐existing lipid domains or induces the formation of specialized membrane domains. Recent evidence suggests that Gag assembles PI(4,5)P_2_/Chol nanoclusters that exclude SM in symmetric model membranes, indicating that Gag does not directly interact with SM [109].

**FIGURE 4:**
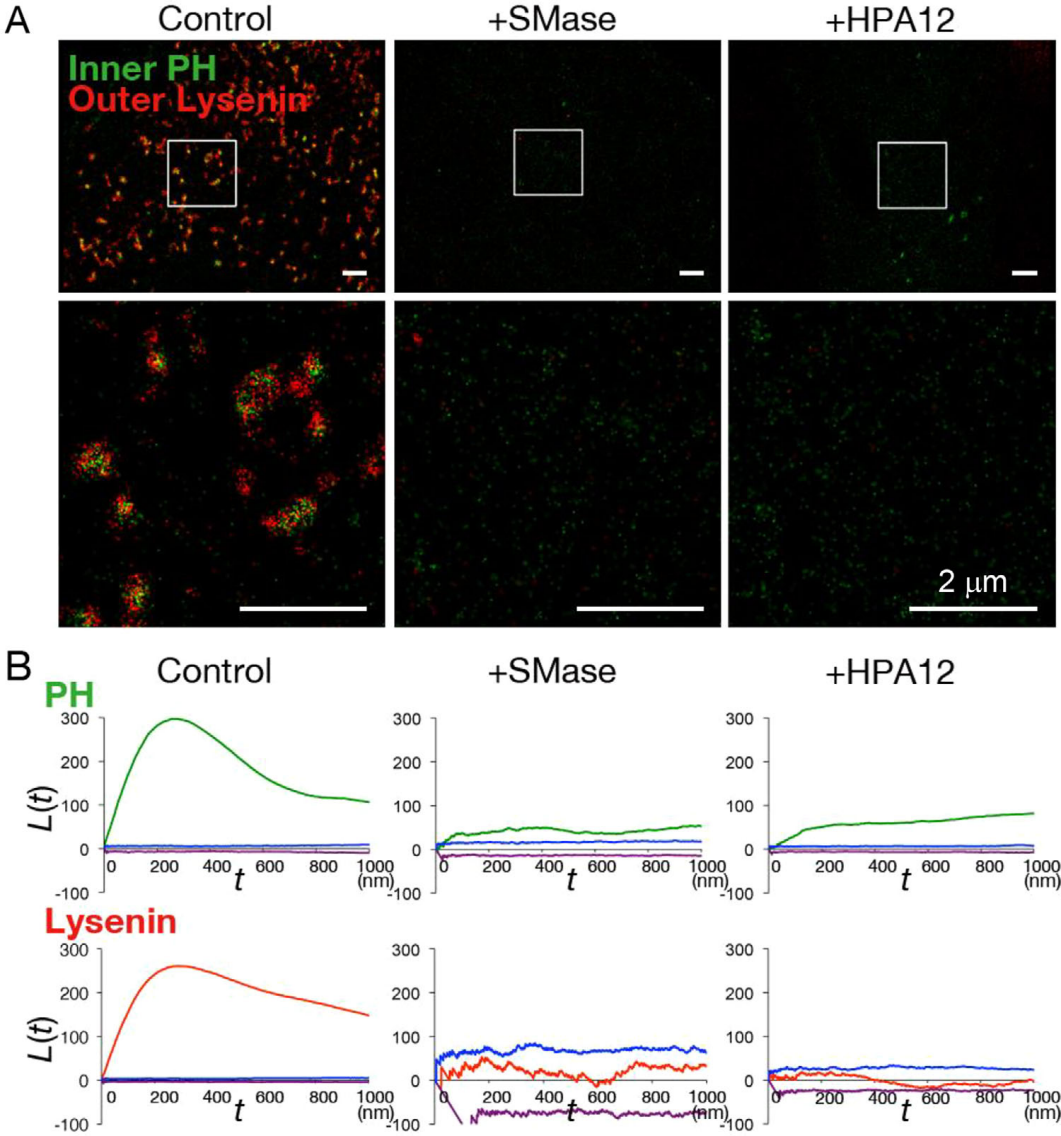
Transbilayer colocalization between SM‐rich domains in the outer leaflet and PI(4,5)P_2_‐rich domains in the inner leaflet of the plasma membrane. **(A)** PALM/dSTORM images of Dronpa‐PH (green) and AF647‐NT‐Lys (red). LLC‐PK1 cells expressing Dronpa‐PH were labeled with AF647‐NT‐Lys. **(Center)** Cells treated with bacterial sphingomyelinase (SMase) for 1 h. **(Right)** Cells treated with HPA12 for 48 h. **(Lower panels)** Magnified views of the regions surrounded by white squares in the upper panels. **(B)** Ripley's L‐function analysis [112, 113, 114] of Dronpa‐PH (green) and AD647‐NT‐Lys (red) distributions. L(t) > 0 indicates clustering; L(t) < 0 indicates dispersion, and L(t) = 0 indicates a random distribution. Blue and magenta lines represent the upper and lower bounds of the 99% confidence envelope, respectively. L(t) values above the envelope indicate statistically significant clustering. Adapted from [110].

Previously, we investigated the transbilayer co‐localization of SM‐ and PI(4,5)P_2_‐rich domains using PALM/dSTORM [110] (Figure 4). To label SM, NT‐Lys was conjugated with Alexa Fluor 647 (AF647‐NT‐Lys). For PI(4,5)P_2_ labeling, the pleckstrin homology (PH) domain of human phospholipase Cδ1 (PLCδ1) [111] was fused to the fluorescent protein Dronpa (Dronpa‐PH) and expressed in pig kidney‐derived epithelial cell line, LLC‐PK1 cells. Cells expressing Dronpa‐PH were incubated with AF647‐NT‐Lys to simultaneously label SM.

PALM/dSTORM imaging revealed the co‐localization between SM‐rich domains in the outer leaflet and the PI(4,5)P_2_‐rich domains in the inner leaflet of the PM [110] (Figure 4A,B). Reduction of SM by either enzymatic digestion with exogenous sphingomyelinase (SMase) or inhibition of SM biosynthesis using HPA‐12 abolished outer leaflet SM‐rich domains (Figure 4A,B). Notably, depletion of SM also led to the dispersion of PI(4,5)P_2_‐rich domains in the inner leaflet (Figure 4A,B). In contrast, reduction of PI(4,5)P_2_ did not affect the organization of SM‐rich domains [110]. Importantly, exogenous addition of SM rescued both SM and PI(4,5)P_2_ domain organization following SM depletion. Chol depletion also abolished SM domains [110].

Together, these findings demonstrate that: (1) PI(4,5)P_2_ forms lipid domains in the inner leaflet of the PM, underlying SM‐rich domains in the outer leaflet; (2) Chol is essential for the formation of SM‐rich domains; and (3) SM‐rich domain in the outer leaflet regulates the formation and maintenance of PI(4,5)P_2_ domains in the inner leaflet.

A subsequent study using *nakanori*, a probe that selectively binds SM‐ and Chol‐rich domains, further confirmed the transbilayer co‐localization of SM‐, Chol‐, and PI(4,5)P_2_‐rich domains [66].

## 5 Possible Molecular Mechanisms of Transbilayer Co‐Localization of SM‐Rich Domains and PI(4,5)P_2_‐Rich Domains

**FIGURE 5:**
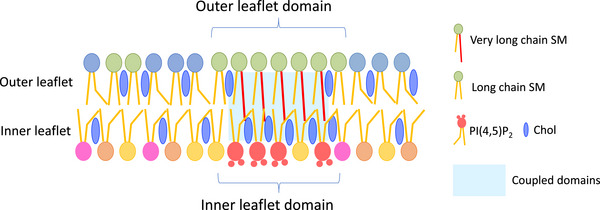
Model 1: Interdigitation‐mediated transbilayer interaction between SM‐rich domain and PI(4,5)P_2_‐rich domain. Very long acyl chains (C22–C24) of SM in the outer leaflet of the plasma membrane extend into the inner leaflet, allowing physical coupling with saturated acyl chains (e.g., C18:0 stearic acid) of PI(4,5)P_2_. This interdigitation facilitates the spatial co‐localization of SM‐rich domains in the outer leaflet with PI(4,5)P_2_‐rich domains in the inner leaflet. Chol, enriched in both leaflets, may further stabilize this transbilayer organization by modulating lipid packing and domain order.

One proposed mechanism for coupling lipid domains across the bilayer involves the interdigitation of very long acyl chains (C22–C24) of sphingomyelin (SM) into the opposing leaflet, allowing direct interaction with the acyl chains of inner leaflet lipids [70, 115, 116] (Figure 5, model 1). As an inner leaflet lipid, stearic acid (C18:0)‐containing PI(4,5)P_2_ is postulated [13, 57, 88]. Conversely, Hornor et al. [117] suggested that tail interdigitation alone cannot explain interleaflet coupling using symmetric fluid diphytanoyl phosphatidylglycerol (DPhPG) membranes and fluorescent lipidic dyes with different acyl chain lengths (F2N12S and DiIC or DiOC; C12 and C18, respectively). Nevertheless, in asymmetric liposomes lacking Chol, outer leaflet very long chain SM, but not long chain (C18) SM, was shown to induce ordered domains in the inner leaflet [104]. Supporting these findings, atomistic molecular dynamics (MD) simulations of asymmetric membranes containing Chol have suggested that interdigitation of very long chain SM can drive interleaflet coupling, with Chol modulating lipid order in the cytosolic leaflet [118].

The fatty acid composition of SM in cells and PMs varies among cell types. Both long chain and very long chain SM species are incorporated into the HIV‐1 viral envelope. Enrichment of specific SM molecular species in HIV relative to the PM is cell‐type dependent and varies based on methods used to isolate PM fractions [11, 12, 13]. For example, in virions derived from HeLa P4 cells, C16:0 SM was slightly increased and C24:1 SM slightly decreased relative to the PM [12]. In MT4 human T cell line, C16:0 SM levels were slightly increased or unchanged depending on the purification method [11, 12, 13]. In HIV‐1 produced from H9 human T cell line and monocyte‐derived macrophages (MDM), C16:0 SM and C24:1 SM were slightly increased, while C24:0 SM was reduced [11]. A model membrane study suggested that C16:0 and C24:0 SM species can coexist within the same leaflet [119].

SM acyl chain is determined by a family of six ceramide synthases (CERS1‐6) in mammalian cells, each with substrate specificity for different acyl‐CoA chain lengths. The CERS2 gene encodes the enzyme responsible for the synthesis of very long chain ceramides. Knockout of CERS2 (CERS2 KO) significantly reduces the cellular levels of very long chain SM. Interestingly, CERS2 KO did not impair HIV‐1 assembly or incorporation of the viral envelope protein (Env) into virions, but it did reduce the infectivity of HIV‐1 particles produced in HEK293T cells [2]. Since vesicular stomatitis virus glycoprotein (VSV‐G)‐pseudotyped virions produced in CERS2 KO cells retained full infectivity, the authors concluded that function of Env in membrane binding and/or fusion is dependent on sphingolipid composition [2]. However, the lipid composition of the viral envelopes in this study was not analyzed.

Although very long chain SM is a strong candidate for mediating interleaflet coupling via interdigitation, this mechanism alone does not account for the specific co‐localization between SM‐rich domains and PI(4,5)P_2_‐rich domains. Notably, stearic acid (C18:0)—a saturated fatty acid implicated in this interaction—is also present in other inner leaflet lipids, such as phosphatidylethanolamine (PE) [3, 11, 12, 13, 120, 121], suggesting that C18:0 alone is insufficient to explain the specificity of PI(4,5)P_2_ involvement.

Moreover, the physical properties of SM species with varying acyl chain lengths differ significantly. Very long chain SM (e.g., C24:0) has a lower affinity for Chol compared to long chain SM (e.g., C16:0) [74, 122]. In Chol‐containing model membranes, C24:0 SM fails to form SM‐ and Chol‐rich domains within the same leaflet [123]. Nevertheless, in asymmetric liposomes with Chol, both long and very long chain SM species in the outer leaflet can induce ordered domain formation in the inner leaflet composed of diC18:1 PC, albeit to different extents [124].

**FIGURE 6:**
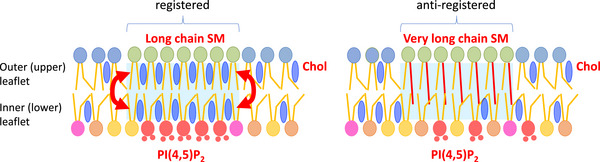
Model 2: Chol‐mediated transbilayer interaction between SM‐rich domain and PI(4,5)P_2_‐rich domain. **(Left)** In membranes enriched with long chain SM (e.g., C16:0 SM), Chol preferentially associates with SM in the outer leaflet, leading to local accumulation. Red arrows indicate the flip‐flop of Chol between the leaflets, where it contributes to the stabilization and clustering of PI(4,5)P_2_‐rich domains. This process facilitates spatial alignment (registration) of liquid‐ordered (Lo) domains across the bilayer. Light blue rectangles highlight bilayer regions with potential leaflet registration. **(Right)** In contrast, outer leaflet domains enriched in very long chain SM (e.g., C24:0 SM), which have lower affinity for Chol, are less capable of recruiting and flip‐flopping Chol. As a result, Chol‐mediated stabilization and clustering of PI(4,5)P_2_ in the inner leaflet are diminished, and interleaflet domain registration is disrupted.

An alternative, cholesterol‐centered mechanism has been proposed (Figure 6, Model 2). Coarse‐grained MD simulations suggest that Chol flip‐flop between leaflets facilitates the transbilayer alignment of Lo domains [125]. Chol is also known to stabilize PI(4,5)P_2_ domains [126, 127], and its spontaneous transbilayer flip‐flop occurs on the millisecond timescale [62, 128]. In Model 2, C16:0 SM associates strongly with Chol in the outer leaflet, promoting local Chol enrichment within SM‐rich domains. At the same time, Chol flip‐flop delivers Chol to the inner leaflet, where it can enhance the stability and clustering of PI(4,5)P_2_ domains (Figure 6, model 2).

Supporting this model, coarse‐grained MD simulations of asymmetric bilayers composed of C16:0 SM:diC18:1 PC:Chol (1:1:1, upper leaflet) and diC16:0 PC:diC18:1 PC:Chol (1:1:1, lower leaflet) clearly showed domain formation in the lower leaflet [129]. Importantly, liquid‐ordered (Lo) domains in both leaflets were *registered*, that is, the domains in opposing leaflets were spatially aligned and showed a similar lipid phase. This is also called phase symmetry. In contrast, when the outer leaflet contained C24:0 SM instead of C16:0 SM, smaller Lo domains formed in the inner leaflet, and the domains between the two leaflets were not registered. Instead, *anti‐registered* (phase asymmetric) domains were observed, in which the lipid phases of the upper and lower leaflets differed [129].

These findings support Model 2, where Chol flip‐flop and Chol‐mediated stabilization play central roles in coupling lipid domains across the bilayer. Nevertheless, the potential involvement of proteins in domain registration cannot be ruled out, as suggested by previous theoretical and computational studies [130, 131].

Gag has been shown to induce the formation of PI(4,5)P_2_‐enriched lipid nanoclusters in model membranes [109, 132], particularly in those enriched with Chol [109]. This clustering activity depends on Gag multimerization: a multimerization‐deficient Gag mutant (W316A/M317A; referred to as Gag‐WM) fails to promote PI(4,5)P_2_ clustering [132]. Similarly, mutations in the matrix (MA) domain that disrupt PI(4,5)P_2_ binding (K30E/K32E) also abolish clustering [132]. These findings indicate that Gag multimerization, together with specific interactions between the MA domain and PI(4,5)P_2_, is required for lipid nanodomain formation.

In vitro liposome experiments demonstrate that Gag preferentially binds to membranes mimicking the Ld phase [109, 132, 133], likely due to the need for insertion of the N‐terminal myristoyl group of Gag into the inner leaflet of the PM. Single‐particle tracking experiments have shown that Gag monomers or low‐order oligomers, after initial targeting to the inner leaflet, are laterally recruited to pre‐existing or nascent assembly sites [134, 135, 136, 137, 138]. It is estimated that each viral particle incorporates approximately 2400 Gag molecules and 7800 PI(4,5)P_2_ molecules [13, 83]. Gag multimerization‐dependent PI(4,5)P_2_ clustering suggests that Gag induces the formation of large PI(4,5)P_2_‐enriched domains in the inner leaflet during assembly.

In addition to lipid interactions, the cortical F‐actin cytoskeleton—located just beneath the inner leaflet—has been proposed to restrict the lateral diffusion of lipids and proteins within the PM [139]. Interestingly, a recent study reported that Gag assembly occurs preferentially in regions depleted of cortical F‐actin. Gag achieves this spatial regulation by recruiting Arpin, an inhibitor of Arp2/3‐mediated actin branching, to the assembly site in infected T cells [140]. This suggests that Gag not only organizes lipids but also modulates the local cytoskeletal environment to facilitate viral assembly.

## 6 Effect of Membrane Curvature on Lipid Selection During Gag Assembly

During HIV‐1 assembly, the multimerizing Gag lattice imposes intrinsic membrane curvature at the budding site [82, 83]. To investigate how this curvature influences lipid domain organization and selection, the behavior of lipid‐binding probes, NT‐Lys and D4, was analyzed using super‐resolution microscopy in cells expressing Gag mutants defective in curvature formation [58]. The Gag‐P99A and EE (E75A E76A) mutants carry point mutations within the N‐terminal domain of CA [141, 142] (Figure 1B) and are known to form relatively flat lattices upon multimerization [57, 85, 143]. In addition, we utilized the Gag‐∆L mutant, which is defective in recruiting the ESCRT complex, a host machinery essential for membrane scission and virion release [57, 85, 144]. Although the Gag‐∆L mutant does not prevent PM curvature changes, it results in viral particles remaining tethered to the cell surface.

PALM/dSTORM imaging revealed that SM‐rich domains formed in cells expressing Gag‐P99A and EE mutants were smaller than those formed by Gag WT. The extent of co‐localization between Gag and SM‐rich lipid domains in these mutants was intermediate between that observed for Gag WT and the WM mutant. Furthermore, FLIM‐FRET analysis of SM‐rich and Chol‐rich domains demonstrated that fluorescence lifetimes and amplitudes in the presence of Gag‐P99A and EE mutants were not significantly different from those in control cells lacking Gag expression. In contrast, both Gag‐∆L and Gag WT produced similar phenotypes with respect to lipid domain formation and protein clustering. These results suggest that the membrane curvature generated by the Gag lattice is critical for efficient domain remodeling, while the ESCRT machinery is not directly required for this process.

The smaller Gag domain sizes observed with curvature‐deficient mutants (Gag‐P99A and EE) were comparable to those seen with multimerization‐deficient mutant (WM). This result suggests that membrane curvature promotes the formation of higher‐order Gag multimers, which is consistent with MD simulations [137].

The formation of membrane curvature is also facilitated by the host factor IRSp53, a member of the I‐BAR domain protein family [145]_._ I‐BAR domains are known to both sense and generate negative membrane curvature and to bind PI(4,5)P_2_ specifically via electrostatic interactions with their positively charged surfaces [146, 147]. These findings point to a cooperative mechanism in which membrane curvature, lipid composition, and host cytoskeletal factors collectively regulate the spatial organization of Gag assembly and viral budding.

Multiple mechanisms have been proposed for membrane curvature generation by both lipids and proteins [148, 149, 150]. Lo domains composed of SM and Chol have been shown to be thicker—and therefore mechanically stiffer—than Ld domains containing, for example, unsaturated PC, as demonstrated by atomic force microscopy (AFM) measurements [151, 152] and bending modulus determinations [153]. In the context of HIV‐1 assembly, it is intriguing that these thicker and stiffer SM‐ and Chol‐rich domains, which require greater bending energy, are nonetheless enriched at the highly curved viral assembly sites.

When highly curved membrane tubes were produced by pulling from GUVs composed of a ternary mixture of SM, Chol, and unsaturated PC, enrichment of Ld domains in the tubes occurred only when the tube radii were below 30–40 nm and the mixture exhibited phase‐separation [148, 154]. Additionally, the presence of lipid‐clustering proteins induced lipid sorting in liposomes even when the lipid composition was far from phase separation [154]. These findings may explain why SM‐rich and Chol‐rich domains are not excluded from the assembly of relatively large HIV particles (50–75 nm radius), and highlight the critical role of the Gag lattice in lipid enrichment. Alternatively, it has been proposed that Gag functions like a detergent, locally softening the membrane and thereby reducing the energy barrier for membrane bending [155].

During Gag multimerization, the assembly site acquires positive curvature at the outer leaflet membrane. This curvature may induce local changes in the membrane hydration of that leaflet. In regions of curvature, the hydrophobic mismatch between lipid domains with different phases—or between long chain and very long chain SM species—can become more pronounced. These mismatches likely increase interfacial hydration at domain boundaries. To minimize this unfavorable hydration, lipids at these boundaries may undergo conformational deformation, contributing to reducing line tension. Thus, line tension at domain boundaries can help to drive membrane curvature growth [156, 157] during Gag lattice assembly.

Experimental insights into these lipid interactions were obtained using heterodyne‐detected vibrational sum‐frequency generation spectroscopy (HD‐VSFG), which probed the structure and hydration of lipid monolayers composed of Chol and either saturated or unsaturated phospholipids [158]. In diC18:1 PC/Chol membrane, Chol did not interact with the diC18:1 PC acyl chain tails and instead intercalated near the membrane interface. This insertion increased acyl chain disorder and the spacing between adjacent diC18:1 PC molecules. In contrast, in the SM/Chol membrane, Chol was found to enhance lipid packing and order the SM tails. In this case, Chol remained embedded deeper in the nonpolar membrane interior.

These differences also affected water molecule organization at the membrane interface. In SM/Chol membranes, water molecules hydrating the choline head groups were more directionally ordered compared to those in diC18:1 PC/Chol membranes. This structured hydration results in a stronger membrane dipole potential in SM/Chol‐rich regions. Under these circumstances, lateral interactions among unsaturated phospholipids become relatively weak, while SM molecules tend to self‐associate together with Chol and preferentially cluster into pre‐existing SM/Chol‐rich domains. These domains are further stabilized by hydrogen bonding networks between SM headgroups and cholesterol.

Taken together, these results suggest that SM/Chol‐rich domains are structurally and energetically favorable in curved regions of the membrane, such as the HIV‐1 assembly site. The strong dipole potential and increased lipid order in these domains likely contribute to their preferential stabilization in the positively curved membrane environment generated by Gag multimerization.

## 7 Conclusion

The HIV‐1 genome does not encode any enzymes involved in lipid metabolism, yet the lipid composition of the viral envelope profoundly influences virion infectivity [2, 3, 4, 5, 6, 7, 9, 109]. This highlights the importance of host lipid interactions during viral assembly at the PM and suggests that targeting these interactions could offer novel therapeutic strategies against HIV‐1. Recent studies have revealed that outer leaflet sphingomyelin (SM)‐rich and cholesterol (Chol)‐rich domains are enriched at the sites of Gag assembly [57, 58, 88]. However, the molecular mechanisms underlying the selective enrichment of these lipid domains, particularly across the bilayer, remain incompletely understood.

In this review, we explored two lipid‐centric models that may explain the transbilayer co‐localization of lipid domains during HIV‐1 assembly:

- **Model 1: Interdigitation‐mediated coupling**, where very long acyl chains of SM in the outer leaflet physically interact with inner leaflet lipids at the Gag assembly site.
- **Model 2: Chol‐mediated coupling**, in which rapid flip‐flop of Chol stabilizes and links outer leaflet SM‐rich domains with inner leaflet PI(4,5)P_2_‐rich domains.

Although both mechanisms sound plausible, our experimental data—alongside published imaging, biochemical, and simulation studies—support Model 2 as the more likely mechanism for Gag‐mediated lipid domain remodeling.

Future studies will aim to test these models through genetic manipulation of fatty acid composition in PM lipids, coupled with high‐resolution lipid imaging and biophysical measurements of local membrane properties. Understanding these mechanisms in greater detail will be essential not only for elucidating the fundamentals of viral assembly but also for identifying new intervention points in the HIV‐1 life cycle.

## Author Contributions

N.T.: Writing original draft, figure design, and editing. Y.M.: Writing and editing. T.K.: Writing original draft, figure preparation, and editing.

## Conflicts of Interest

The authors declare no conflict of interests.

## Data Availability

Any additional information required to reanalyze the data reported in this paper is available from the lead contact upon request.
